# Reliability of magnetic resonance imaging findings in symptomatic minor instability of the lateral elbow: A cross‐specialty evaluation

**DOI:** 10.1002/jeo2.70833

**Published:** 2026-07-22

**Authors:** Ana Catarina Ângelo, Aline Serfaty, Diana Afonso, Francesca Stefanini, Clara de Campos Azevedo

**Affiliations:** ^1^ Joaquim Chaves Saúde I Hospital dos SAMS de Lisboa, Shoulder and Elbow Unit Orthopedics and Traumatology Lisbon Portugal; ^2^ Medscanlagos Radiology Cabo Frio Rio de Janeiro Brazil; ^3^ Departamento de Radiologia, Hospital CUF Tejo Unidade de Imagem Musculoesquelética Lisbon Portugal; ^4^ Arcispedale Sant′Anna ‐ University Hospital of Ferrara, Orthopaedics and Traumatology Unit Ferrara Italy

**Keywords:** elbow pain, epicondylitis, interobserver agreement, lateral elbow instability, magnetic resonance imaging, symptomatic minor instability

## Abstract

**Purpose:**

Symptomatic minor instability of the lateral elbow (SMILE) was recently described as an atraumatic or microtraumatic (overuse) cause of lateral elbow pain. The purpose of this study was to describe magnetic resonance imaging findings in arthroscopically confirmed SMILE and evaluate the reproducibility of predefined imaging features. It was hypothesised that standard MRI could identify recurrent structural findings associated with SMILE, although variability in interobserver agreement was expected.

**Methods:**

Patients who underwent elbow arthroscopy for the treatment of atraumatic recalcitrant lateral elbow pain between 2021 and 2024 by the same surgical team were retrospectively included in this study. Inclusion criteria were arthroscopic identification of a minimum of three signs of minor instability, and available preoperative MRI images. The preoperative MRI images were independently evaluated by five raters (three orthopaedic surgeons and two specialised musculoskeletal radiologists). Prevalence, inter‐rater reliability, intra‐ and inter‐group agreement were calculated for each finding. The significance level was set at *p* = 0.05.

**Results:**

Thirteen patients were included in the study. The kappa coefficient values ranged from 0.036 (95% confidence interval [CI], −0.14 to 0.21) to 0.412 (95% CI, 0.24 to 0.58), indicating fair to moderate agreement across findings. Common extensor partial tear in Coronal T2 or PD FS was the only considered both prevalent and reliable.

**Conclusion:**

MRI findings in arthroscopically confirmed SMILE demonstrated variable interobserver reproducibility, with only selected features showing acceptable agreement. While standard MRI may identify structural changes associated with SMILE, the limited reproducibility of several findings highlights the need for standardised imaging criteria and further investigation, including advanced imaging techniques such as intra‐articular imaging techniques.

**Level of Evidence:**

Level III, retrospective diagnostic reliability study.

## Graphical abstract

Standard magnetic resonance imaging identified recurrent findings associated with symptomatic minor instability of the lateral elbow, although most instability‐related features demonstrated limited interobserver reproducibility.

**Abbreviations**
CI: confidence interval
CT: computed tomography
Ex: examiner (e.g., Ex1, Examiner 1)
FS: fat‐saturated
MRI: magnetic resonance imaging
Na: not applicable
PD: proton density
R‐LCL: radial collateral ligament
SMILE: symptomatic minor instability of the lateral elbow
UH joint: ulnohumeral joint

## INTRODUCTION

Symptomatic minor instability of the lateral elbow (SMILE) is a recently recognised condition characterised by atraumatic, persistent lateral elbow pain related to prolonged repetitive varus/pronation‐stress during daily activities [3, 4, 7]. Interest in SMILE has grown in recent years due to its association with recalcitrant chronic lateral elbow and persistent mechanical symptoms despite standard conservative treatment [2, 13, 14, 15].

The pathophysiology behind SMILE highlights the structural and functional significance of the radial band of the lateral collateral ligament (R‐LCL) as a primary stabiliser during varus loading with the elbow flexed between 50° and 70° [3, 4, 7, 18]. In an anatomical cadaveric study, Arrigoni and colleagues demonstrated that elongation and patholaxity of the R‐LCL can lead to progressive insufficiency of the annular ligament, resulting in abnormal motion of the radial head within the lateral compartment of the elbow and dynamic incongruence of the proximal radioulnar joint [5]. In a case series with arthroscopic evaluation, the same authors reported that over 45% of patients with recalcitrant lateral epicondylitis demonstrated at least one of the following three signs of lateral ligamentous patholaxity: annular drive through, loose collar sign and laxity of the radial component of the R‐LCL; and more than 85% had intra‐articular findings such as anterior radio‐ulnar synovitis, anterolateral capsular tear, or chondropathy of the radial head and capitellum [3].

Diagnosing SMILE is challenging due to symptom overlap with other lateral elbow pain‐related conditions. Patients with SMILE typically report nonspecific complaints that can overlap with those associated with lateral epicondylitis, radial tunnel syndrome and low‐grade posterolateral rotatory instability (PLRI), which may easily lead to misdiagnosis and inadequate treatment [3, 7, 21, 22]. Recent research emphasises the role of dynamic imaging techniques, particularly musculoskeletal ultrasound, in detecting subtle lateral elbow instabilities that may be missed during physical examination [6, 9, 16].

The SMILE index, a semiquantitative scoring system based on CT arthrography findings was developed to identify radiologic features associated with minor instability, including asymmetrical joint space widening, annular ligament laxity, synovial thickening and chondral wear patterns on the radial head and capitellum [21]. Magnetic resonance imaging (MRI) is a non‐invasive, reproducible modality that provides detailed evaluation of the soft tissue structures associated with SMILE, including the LCL complex, annular ligament and anterolateral capsule and also detect intra‐articular pathology, such as chondral lesions or joint effusion [1, 8, 10, 19, 20].

The purpose of this study was to describe MRI findings in patients with arthroscopically confirmed SMILE and to evaluate the reproducibility of predefined imaging features across a cross‐specialty panel of observers. It was hypothesised that standard MRI could identify recurrent structural findings associated with SMILE, with variability in interobserver agreement given the subtle nature of this condition.

## MATERIALS AND METHODS

### Ethics

This study was approved by the local ethics committee and given the IRB approval number 2006/01. The need for informed consent was waived by the ethics committee.

### Study design

**Figure 1:**
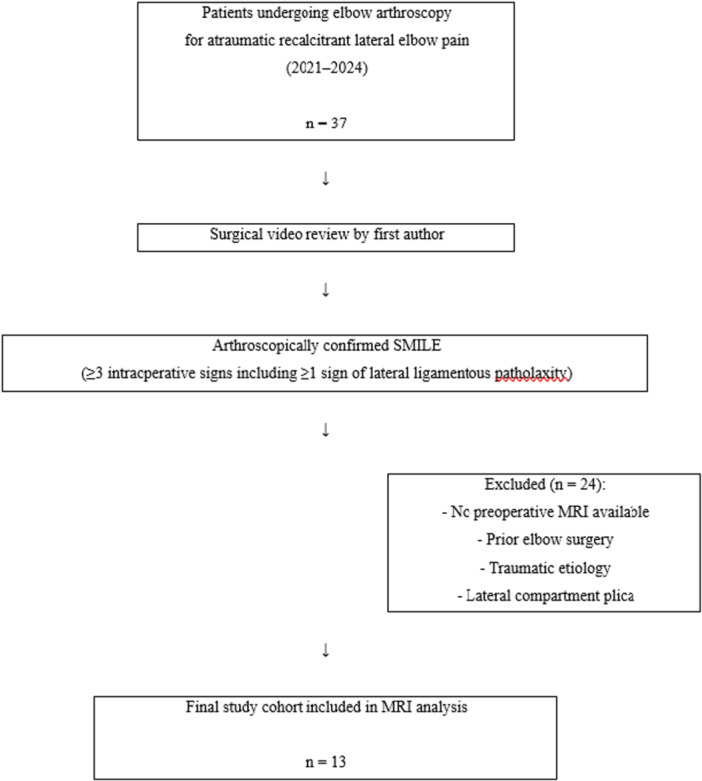
Flow diagram illustrating patient screening and inclusion process. MRI, magnetic resonance imaging; SMILE, symptomatic minor instability of the lateral elbow.

Thirty‐seven patients who underwent elbow arthroscopy for atraumatic recalcitrant lateral elbow pain between 2021 and 2024 by the same surgical team (A.C.A. and C.A.) were retrospectively screened for inclusion. Surgical video recordings were reviewed to confirm the presence of arthroscopic findings consistent with SMILE, defined as at least three characteristic intra‐articular signs, including one sign of lateral ligamentous patholaxity, as previously described by Arrigoni et al. Patients with persistent lateral elbow pain despite a minimum of 3 months of physiotherapy and available preoperative MRI examinations were included. Patients with traumatic elbow instability, previous elbow surgery, or lateral compartment plica were excluded. The patient screening and inclusion process is summarised in Figure 1.

### Surgical evaluation

**Figure 2:**
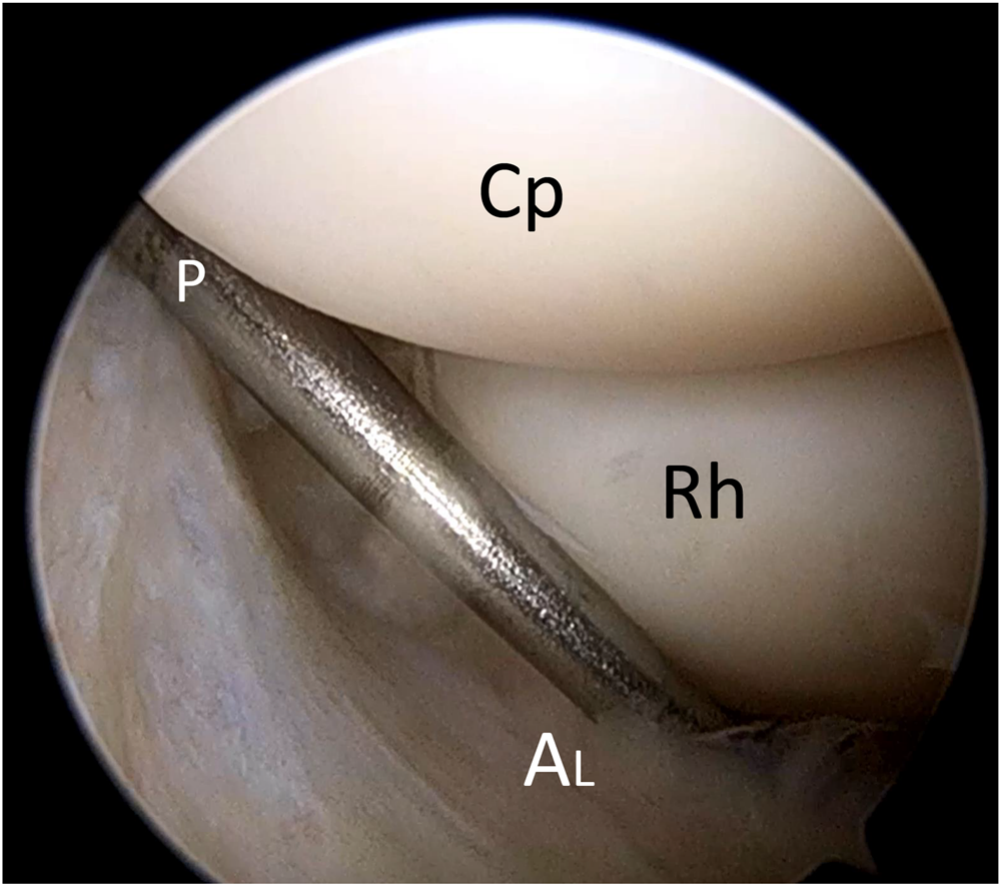
Arthroscopic view of a right elbow using the proximal antero‐medial portal as a viewing portal; The probe (P) is introduced through the direct lateral portal to assess the integrity of the lateral elbow structures; AL, annular ligament; Cp, capitellum; Rh, radial head.

Patients were positioned in lateral decubitus with the operated arm supported by a mechanical arm holder, allowing full flexion and extension of the elbow. Each patient signed an informed consent for the surgical procedure. Approximately 20cc of saline were injected intraarticularly through the soft spot to fill the joint and widen the capsule. A proximal antero‐medial portal was established approximately 2 cm proximal to the medial epicondyle and used as a visualisation portal to approach the anterior compartment of the elbow. A direct lateral portal was established under direct visualisation and used as a working portal (Figure 2). A 4‐mm, 30‐degree arthroscope was used to evaluate the capitellum, radial head, antero‐lateral capsule, R‐LCL, annular ligament and the proximal radio‐ulnar joint (PRUJ). All evident signs of patholaxity and intra‐articular findings previously described as related to SMILE were routinely registered.

### MRI evaluation

MRI studies using 1.5 and 3T magnets were included (Siemens Healthineers, Erlangen, or GE, General Electrics Healthcare, USA). Patients were scanned in the prone position with the arm elevated and elbow pronated (superman position) or supine with the elbow in extension. For inclusion, all studies were required to include conventional multiplanar protocol standard anatomic turbo (fast) spine echo sequences with and without fat‐suppressed sequences. Specifically, T1 and T2 proton density fat suppressed (PD FS) acquisitions, including axial, coronal and sagittal sequences.

**Figure 3:**
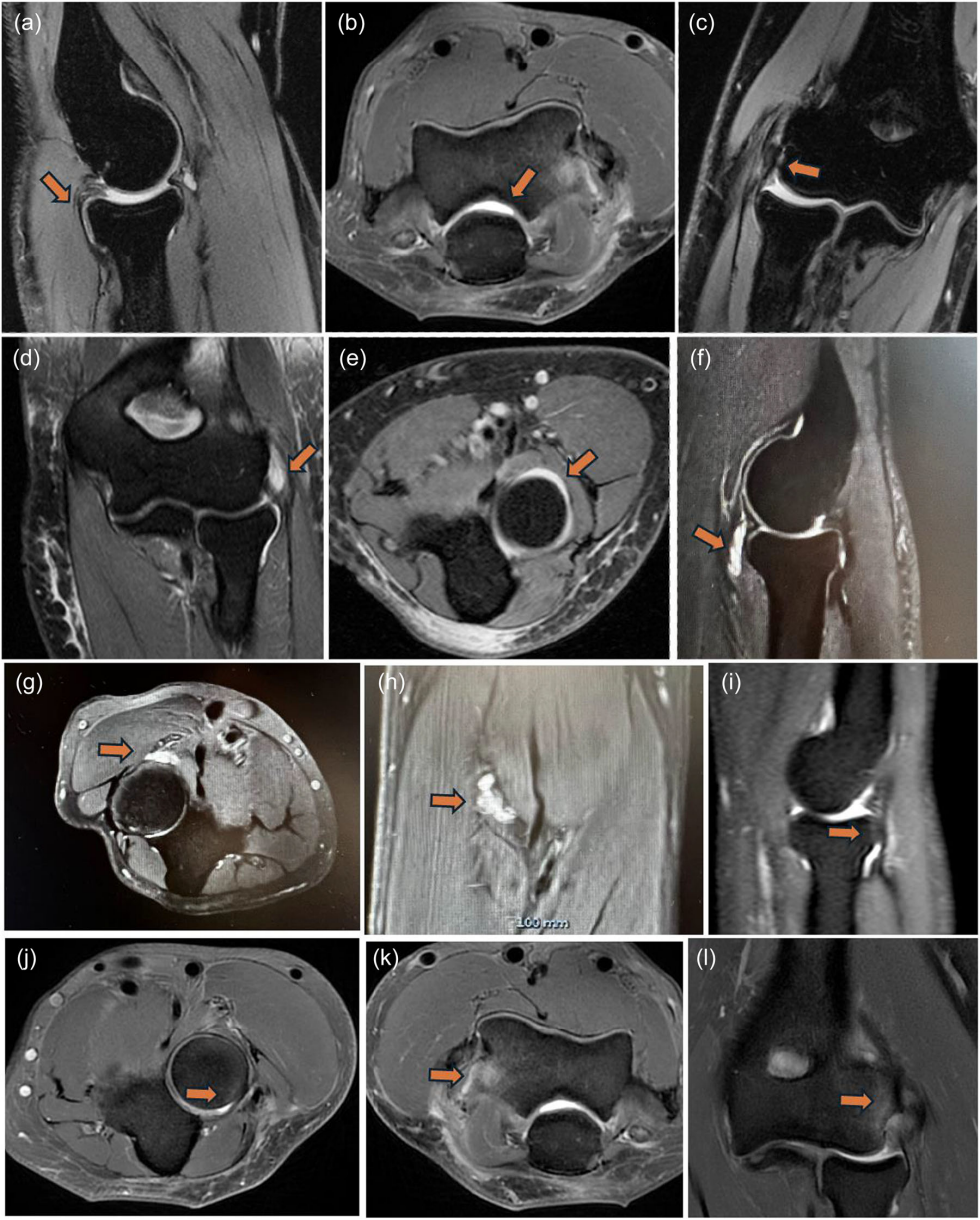
Examples of findings evaluated on the preoperative MRI. (a) Annular ligament sagging (arrow) in a sagittal PD FS image. (b) Lateral minor incongruency (asymmetry) of the ulnohumeral joint (arrow) in an axial PD FS image. (c) R‐LCL proximal tear/discontinuity—L‐sign (arrow) in a coronal PD FS image; the synovial fluid accumulated between the radial collateral ligament (R‐LCL) and the capitellum creates the vertical line, while the radio‐capitellar joint space forms the horizontal line—together resembling the shape of an ‘L; (d) Common extensor partial tear (arrow) in a coronal PD FS image. (e) Annular ligament sagging/fluid around the RH‐Eclipse sign (arrow) in an axial PD FS image. (f) Anterior peri‐annular synovial cyst (arrow) in a sagittal PD FS image. (g) Anterior peri‐annular synovial cyst (arrow) in an axial PD FS image. (h) Anterior peri‐annular synovial cyst (arrow) in a coronal PD FS image. (i) Radial head chondropathy (arrow) in a sagittal PD FS image. (j) Radial head chondropathy (arrow) in an axial PD FS image. (k) Capitellum lateral chondropathy (arrow) in an axial PD FS image. (l) Capitellum lateral chondropathy (arrow) in a coronal PD FS image. FS, fat‐saturated; MRI, magnetic resonance imaging; PD, proton density.

Before the independent readings, the MRI raters participated in a training/calibration session to improve inter‐rater reliability. During this session, representative MRI cases of SMILE (different from those included in the study cohort) were reviewed jointly, and the predefined imaging signs were clarified and standardised (Figure 3).

Each one of the five authors (three orthopaedic elbow surgeons and two specialised musculoskeletal radiologists, each with more than 10 years of experience) independently and retrospectively evaluated the preoperative MRI images. The readers were blinded to the original MRI reports and to each other′s assessments, but not to the fact that all patients had arthroscopically confirmed SMILE.

For every patient, each evaluator was given a standardised table to complete, giving dichotomous answer (yes/no) regarding the presence of predetermined MRI signs (Table 1). All MRI findings were examined in the imaging plane and sequence that best demonstrated its features. Raters were instructed to apply standardised definitions to minimise variability.

**Table 1:** Predetermined findings evaluated in the preoperative MRI images.

Sagittal T2 or PD FS	
Annular ligament sagging	Y/N/Na
RH chondropathy	Y/N/Na
Capitellum lateral chondropathy	Y/N/Na
Anterior peri‐annular synovial cyst	Y/N/Na

Axial T2 or PD FS	
Anterior peri‐annular synovial cyst	Y/N/Na
Lateral minor incongruency (asymmetry) UH joint	Y/N/Na
RH chondropathy	Y/N/Na
Capitellum lateral chondropathy	Y/N/Na
Annular ligament sagging/Fluid around the RH‐ Eclipse sign	Y/N/Na

Coronal T2 or PD FS	
R‐LCL proximal tear/discontinuity (L‐sign)	Y/N/Na
Common extensor partial tear	Y/N/Na
RH chondropathy	Y/N/Na
Capitellum lateral chondropathy	Y/N/Na
Anterior peri‐annular synovial cyst	Y/N/Na

Abbreviations: N, not present; Na, non‐available; PD FS, proton density fat saturation; RH, radial head; R‐LCL, radial band of the lateral collateral ligament; UH, ulno‐humeral; Y, present.

If the required MRI slice was not available, or if the evaluator judge it insufficient to determine the presence of a sign, the finding was classified as non‐available (NA). Prevalence was defined as the proportion of patients classified as positive for each imaging sign according to a majority consensus (≥3 out of 5 raters indicating ‘Yes′). This consensus‐based definition was chosen to reduce the influence of outlier ratings and to reflect agreement across multiple observers, rather than reporting the prevalence according to each rater individually.

### Statistical analysis

Prevalence was calculated for each feature according to the previously mentioned definition. For each MRI finding, Fleiss′ kappa values were calculated to assess the inter‐rater reliability between the five raters and interpreted according to the Landis and Koch criteria: less than 0.2 represents poor agreement; 0.21–0.4 represents fair agreement; 0.41–0.6 represents moderate agreement; 0.61–0.8 represents substantial agreement; >0.8 represents great agreement [17]. Interrater reliability was assessed using the unweighted Fleiss′ kappa coefficient, which is appropriate for nominal dichotomous variables (‘Yes’ or ‘No’) without inherent order [12]. Intra‐group agreement among orthopaedic surgeons (raters 1, 2 and 5, respectively R1, R2 and R5) was determined by calculating the raw concordance between each rater pair (R1–R2, R1–R5 and R2–R5), and averaging the three values. For radiologists (raters 3 and 4, respectively R3 and R4), agreement was determined as the proportion of identical ratings for each feature, excluding missing data. Inter‐group agreement between radiologists and surgeons was assessed by comparing the classification (yes or no) within each group for each patient and MRI feature. A matching majority between groups was defined as concordance. For all analyses, NA ratings were excluded. Prevalence and agreement metrics were calculated based only on cases with valid ‘yes′ or ‘no′ assessments for each finding. A post‐hoc power analysis and sample size calculation was performed based on Bonett′s approximation for detecting agreement beyond chance using Fleiss′ kappa, for a power of 80%. To detect a moderate agreement level (*κ* = 0.6), statistically greater than a null hypothesis value of *κ* = 0.2, with a two‐sided alpha of 0.05, a minimum of 16 subjects was estimated.

The significance level was set at *p* = 0.05. SPSS Statistics Version 27 (IBM) was used for the statistical analysis.

## RESULTS

Of the 37 screened patients, 13 met all inclusion criteria, primarily due to the availability of complete preoperative MRI studies and confirmation of arthroscopic SMILE criteria. The dominant arm was affected in 77% (10/13) of the patients and 23% (3/13) of the patients had a manual job that involved repetitive movements with the affected elbow.

**Figure 4:**
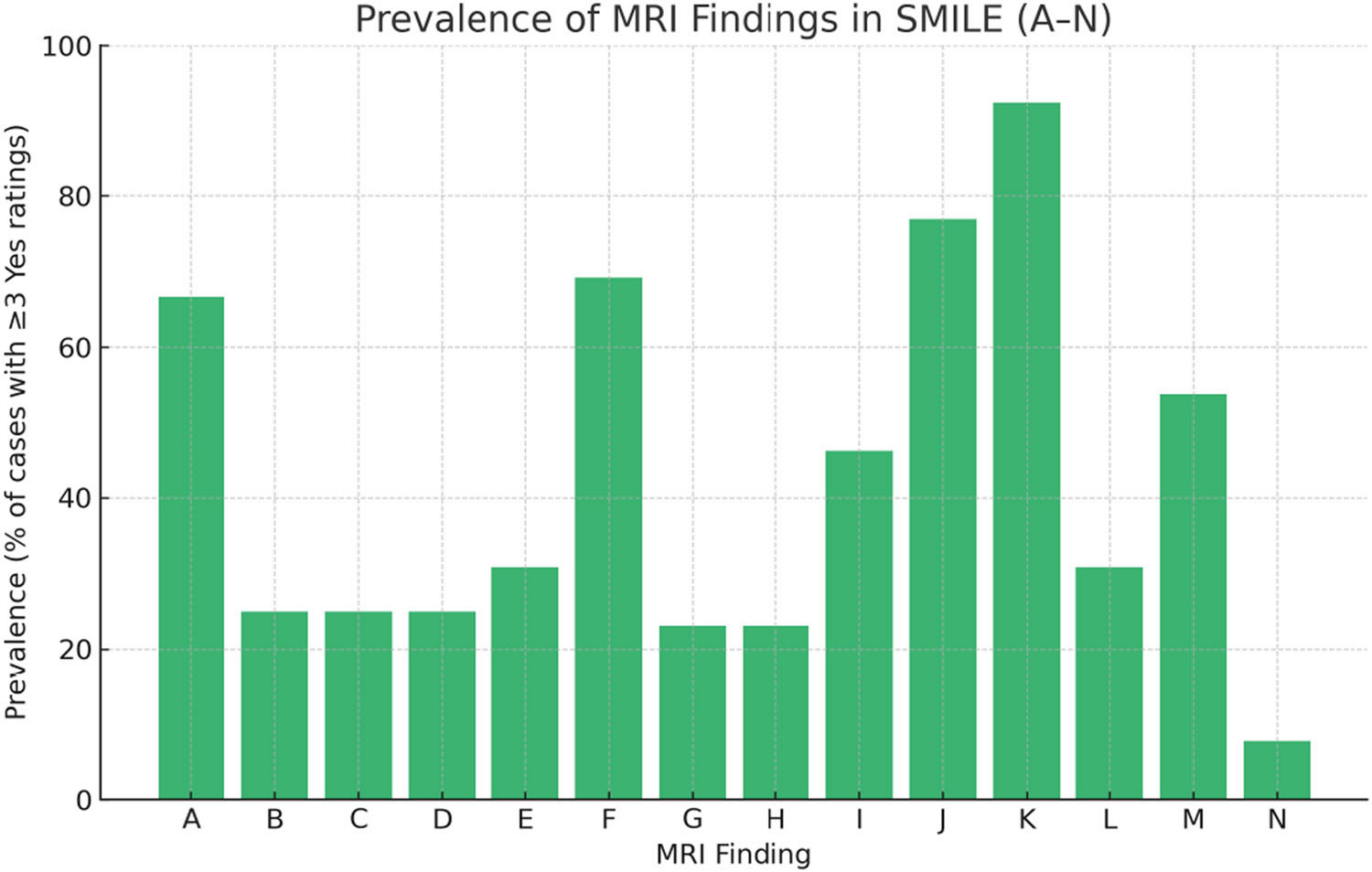
Prevalence of magnetic resonance imaging (MRI) findings evaluated by the five raters. Prevalence was defined as the percentage of patients with a positive score (≥3 of 5 raters indicating ‘Yes′) for each imaging sign. Findings A‐N are specified in Table 1. SMILE, symptomatic minor instability of the lateral elbow.

**Figure 5:**
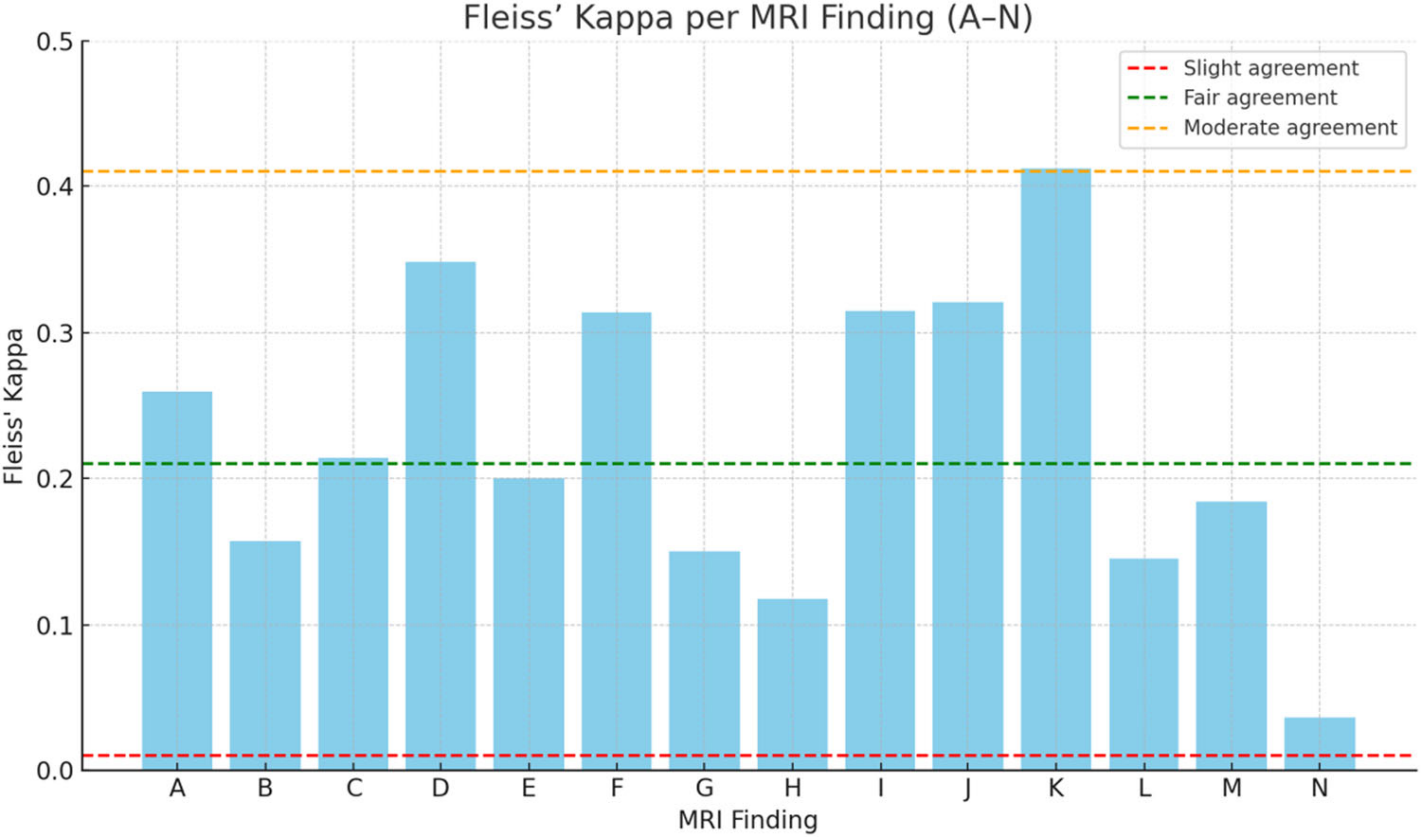
Fleiss′ kappa values for inter‐rater agreement on the evaluated magnetic resonance imaging (MRI) findings. Agreement levels are classified using Landis and Koch criteria: slight (<0.2, above the red dashed line), fair (0.21–0.40, above the green dashed line) and moderate (≥0.41, above the orange dashed line). Findings A‐N are specified in Table 1.

**Figure 6:**
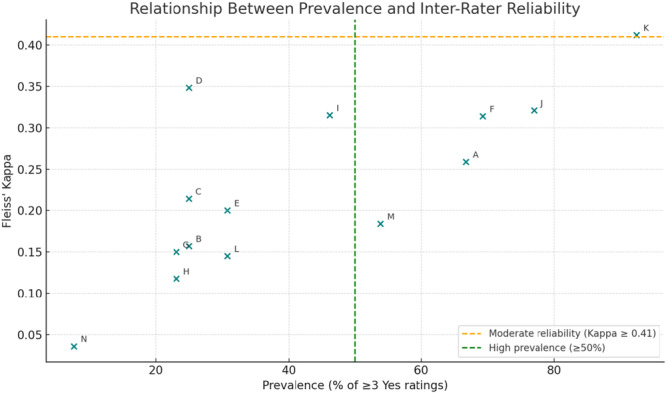
Scatter plot depicting the relationship between prevalence and inter‐rater reliability for each magnetic resonance imaging (MRI) finding. The *X*‐axis represents the prevalence of each finding, defined as the percentage of patients with ≥3 of 5 raters indicating ‘Yes′. The *Y*‐axis represents the Fleiss′ kappa calculated for each finding. Horizontal and vertical dashed lines represent the thresholds for moderate reliability (kappa ≥ 0.41) and high prevalence (≥50%), respectively. Findings in the upper‐right quadrant are considered both prevalent and reliable. Findings A‐N are specified in Table 1.

The more prevalent finding was common extensor partial tear in Coronal T2 or PD FS (K) and features A, F, J, K and M were considered prevalent in more than half of the patients (Figure 4). The kappa coefficients values ranged from 0.036 (95% confidence interval [CI], −0.14 to 0.21) to 0.412 (95% CI, 0.24 to 0.58), indicating a fair to moderate agreement depending on the evaluated feature. The only finding that reached a moderate reliability was common extensor partial tear (Figure 5). Common extensor partial tear was the only finding considered both prevalent and reliable (Figure 6). Intra‐group radiologist raw agreement (R3 and R4) ranged from 91.7% to 100%. Raw agreement between orthopaedic surgeons (R1, R2 and R5) ranged from 38.5% to 84.6%. Inter‐group concordance varied, depending on the evaluated finding, and ranged from 25% ‐ feature M ‐ to 100% ‐ features C, D, E and K. The lowest inter‐group concordance was observed for findings M and A (25.0% and 27.3%, respectively) (Table 2).

**Table 2:** Prevalence, inter‐rater agreement, intra and inter‐group agreement for each magnetic resonance imaging finding.

Finding	Prevalence (≥ 3 yes) (%)	Fleiss′ Kappa (All raters)	Radiologist intra‐group agreement (%)	Orthopaedic surgeon intra‐group agreement%	Inter‐group concordance (%)	95% confidence interval
A	66.7	0.259	91.7	75.0	27.3	0.09–0.43
B	25.0	0.157	100.0	41.7	83.3	−0.01 to 0.33
C	25.0	0.214	91.7	58.3	100.0	0.04–0.39
D	25.0	0.348	91.7	75.0	100.0	0.18–0.52
E	30.8	0.2	92.3	69.2	100.0	0.03–0.37
F	69.2	0.314	100.0	84.6	38.5	0.14–0.49
G	23.1	0.15	100.0	69.2	76.9	−0.02 to 0.32
H	23.1	0.118	92.3	53.8	66.7	0.05–0.29
I	46.2	0.315	100.0	84.6	69.2	0.14–0.49
J	76.9	0.321	100.0	69.2	84.6	0.15–0.49
K	92.3	0.412	100.0	76.9	100.0	0.24–0.58
L	30.8	0.145	100.0	38.5	92.3	−0.03 to 0.32
M	53.8	0.184	92.3	53.8	25.0	0.01–0.36
N	7.7	0.036	92.3	84.6	83.3	−0.14 to 0.21

*Note*: Raters are referred to as R1, R2, R3, R4 and R5. Fleiss′ kappa interpretation was considered according to the Landis and Koch criteria. Fleiss′ kappa represents inter‐rater agreement among all five raters. Radiologist and surgeon agreement columns represent raw intra‐group agreement percentages. Inter‐group concordance represents agreement between the radiologist and surgeon groups.

## DISCUSSION

The most relevant observation from this study is that several MRI features can be identified in patients with arthroscopically confirmed SMILE, although their reproducibility across observers varies. These findings represent an important step toward characterising the imaging spectrum of this emerging clinical entity and provide an initial framework for standardised MRI assessment. While agreement levels differed between imaging signs, the identification of potentially reproducible features suggests that MRI may contribute to the evaluation of atraumatic lateral elbow pain when interpreted within a structured and evolving framework. By being aware of the association between these MRI features and the arthroscopic presence of patholaxity signs in patients with atraumatic recalcitrant lateral elbow pain, both orthopaedic surgeons and specialised radiologists can better diagnose cases of SMILE. Across all the evaluated MRI signs, finding K (common extensor partial tear) demonstrated the highest inter‐rater agreement (Fleiss′ kappa = 0.412, 95% CI: 0.24–0.58). The common extensor origin (CEO), and especially the extensor carpi radialis brevis (ECRB) tendon, acts as a secondary stabiliser to the LCL in varus‐pronation activities. As it was highlighted by Arrigoni and colleagues, ECRB fibres run parallel to the R‐LCL, and in cases of progressive R‐LCL insufficiency, varus‐pronation stability would be attained by overloading the ECRB, leading to its degeneration and tear [3, 11]. This can explain the high prevalence of partial CEO tears seen in these patients. However, partial tearing of the common extensor origin was the only MRI feature demonstrating both prevalence and moderate reproducibility. This finding can be frequently observed in multiple causes of chronic lateral elbow pain and therefore lacks specificity for SMILE. Findings A (annular ligament sagging in sagittal T2 or PD FS), F (lateral minor incongruency of the UH joint in axial T2 or PD FS) and J (R‐LCL proximal tear/discontinuity—L‐sign—in coronal T2 or PD FS) were considered highly prevalent (≥66.7%) and had a fair agreement between raters as indicated by Fleiss′ kappa values with 95% CIs entirely above zero, supporting the statistical significance of the observed agreement beyond chance. These findings are aligned with the pathological cascade of SMILE, in which progressive elongation of the R‐LCL leads to annular ligament sagging and subtle joint incongruence. Finding A (annular ligament sagging in sagittal T2 or PD FS) reflects early soft‐tissue elongation or redundancy, consistent with lateral patholaxity (e.g., loose collar sign and radial head ballottement) documented in arthroscopic studies. Finding F (lateral minor incongruency of the UH joint in axial T2 or PD FS) reflects the subtle misalignment of the ulnohumeral joint resulting from the repetitive varus–pronation stress. Finally, finding J (R‐LCL proximal tear/discontinuity—L‐sign—in coronal T2 or PD FS) is consistent with the structural compromise at the humeral insertion of the R‐LCL, and can be explained by its progressive elongation and laxity and adjacent capsular tear. As only a few findings demonstrated both substantial prevalence and fair to moderate reliability, this might suggest that only selected MRI signs may be robust for clinical use and warrant further prospective validation. Several findings considered more suggestive of lateral ligament patholaxity demonstrated only slight to fair agreement across observers. From a clinical perspective, these results indicate that standard MRI currently has limited reliability as an isolated diagnostic tool for SMILE, particularly when subtle instability‐related findings are evaluated.

Inter‐rater agreement in the interpretation of MRI features differed between radiologists and orthopaedic surgeons. Intra‐group inter‐rater raw agreement differed between radiologists and orthopaedic surgeons, with higher concordance observed among radiologists. This apparent discrepancy must be interpreted cautiously, as the radiologist group included only two raters, while the orthopaedic surgeon group included three. Agreement metrics such as Fleiss′ kappa are influenced not only by the number of raters but also by the distribution of their responses. With only two raters, the estimates are less stable and not directly comparable to those derived from larger groups. Therefore, the observed differences in intra‐group agreement should not be overinterpreted as reflecting true speciality‐specific diagnostic performance, but rather considered a methodological limitation of the study design.

In the SMILE semiquantitative index proposed by Zagarella et al., CT arthrography was used to score features of minor lateral elbow instability, including annular ligament laxity, humeroradial joint incongruity, and capsuloligamentous disruption [21]. In our MRI‐based analysis, structurally analogous signs were identified. These findings were among the most prevalent in our cohort and demonstrated fair inter‐rater agreement. MR arthrography or other intra‐articular contrast imaging techniques may represent a valuable alternative for the imaging assessment of SMILE. Compared with standard MRI, intra‐articular contrast may improve visualisation of subtle capsuloligamentous abnormalities, annular ligament insufficiency, and early joint incongruence. Therefore, future comparative studies evaluating standard MRI, MR arthrography, and CT arthrography may help determine the most reliable imaging modality for the assessment of SMILE. With the evolution of imaging protocols, dynamic and/or stress MRI may provide an increased sensitivity and improved anatomical detail, further contributing to the diagnostic algorithm of elbow minor instability, although these techniques are still rarely performed in clinical practice.

This study has several limitations, the more important one being the relatively small sample size of 13 patients. Even though the cohort was well‐defined and all patients had arthroscopically confirmed signs of patholaxity, the limited number of subjects reduces the statistical power to detect moderate to substantial levels of interobserver agreement with confidence. Based on the performed power analysis, a minimum of 16 patients would be required. Consequently, our findings should be interpreted with caution and considered hypothesis‐generating. Future prospective studies with larger sample sizes are needed to confirm the diagnostic accuracy of these imaging features. Another important limitation of this study is the heterogeneity of MRI acquisition. The evaluated exams were performed across multiple imaging centres, using different MRI machines, field strengths, and protocols. While all sequences included standard fat‐suppressed T2 or proton density axial, coronal, and sagittal planes, variations in technical parameters and coil positioning could have influenced both the assessment of subtle features and the observed levels of agreement. This heterogeneity should be considered when extrapolating our findings. Nonetheless, the diversity in imaging conditions can also reflect a realistic clinical variability and strengthen the external validity of our findings. An important limitation of this study is the absence of a control group. Because all included patients had arthroscopically confirmed SMILE, the present design does not allow assessment of specificity or diagnostic performance of the evaluated MRI features. Prevalence within a selected cohort of confirmed cases should therefore not be interpreted as evidence of diagnostic validity. The aim of this investigation was not to establish MRI as a definitive diagnostic tool, but rather to explore the reproducibility and descriptive spectrum of proposed imaging findings in confirmed SMILE. Nonetheless, future studies incorporating appropriate comparator groups—such as patients with lateral epicondylitis without instability, PLRI, or asymptomatic controls—will be essential to determine the true diagnostic utility and specificity of these MRI features.

This study has also several strengths. To the best of the authors′ knowledge, this is one of the first studies to systematically assess static MRI for identifying imaging features associated with SMILE. The inclusion of raters from two distinct clinical specialties allows for cross‐specialty concordance analysis, providing translational insight into both radiologic and surgical perspectives. The evaluation of 14 specific MRI findings enabled a thorough analysis of prevalence and inter‐rater reliability. Lastly, by drawing direct comparisons with the validated CT‐based SMILE index, this study strengthens the case for using static MRI to identify early structural signs of lateral ligament patholaxity in routine practice. Rather than establishing definitive diagnostic criteria, this study aims to open the door to more robust investigations focused on refining MRI definitions, improving reliability, and clarifying the role of imaging in the diagnostic pathway of SMILE. Moving forward, prospective studies with larger cohorts and standardised MRI protocols will be essential to validate which MRI features are both prevalent and reproducible enough to serve as reliable diagnostic markers.

## CONCLUSION

While standard MRI may identify structural changes associated with lateral ligament patholaxity, the limited reproducibility and specificity of several findings currently restrict its reliability as an isolated diagnostic tool for SMILE. These results highlight both the potential and current limitations of MRI in the evaluation of atraumatic lateral elbow pain and emphasise the need for increased awareness of this condition among orthopaedic surgeons and musculoskeletal radiologists. Nevertheless, this study contributes to the characterisation of MRI features associated with this emerging condition and highlights the need for standardised imaging definitions, optimised imaging protocols and further investigation using advanced imaging techniques such as MR arthrography or other intra‐articular imaging techniques.

## AUTHOR CONTRIBUTIONS

Ana Catarina Ângelo and Clara de Campos Azevedo contributed to study conception and design. Ana Catarina Ângelo performed data acquisition and analysis. All authors independently evaluated the pre‐selected MRI scans and recorded the presence or absence of the predefined imaging features used in the study. Ana Catarina Ângelo interpreted the data. Ana Catarina Ângelo drafted the manuscript. All authors revised the manuscript critically, contributed for important intellectual content and approved the final version.

## FUNDING INFORMATION

The authors have no funding to report.

## CONFLICT OF INTEREST STATEMENT

The authors declare no conflicts of interest.

## ETHICS STATEMENT

This study was approved by the Comissão de ética do Hospital dos SAMS de Lisboa, IRB approval number 2006/01. This retrospective study involved only anonymized imaging data and did not require direct patient contact.

## Data Availability

The datasets are available from the corresponding author upon request.
